# γδ T Cell-Mediated Immune Responses in Disease and Therapy

**DOI:** 10.3389/fimmu.2014.00571

**Published:** 2014-11-10

**Authors:** T. Sree Latha, Madhava C. Reddy, Prasad V. R. Durbaka, Aparna Rachamallu, Reddanna Pallu, Dakshayani Lomada

**Affiliations:** ^1^Department of Genetics and Genomics, Yogi Vemana University, Kadapa, India; ^2^Department of Biotechnology and Bioinformatics, Yogi Vemana University, Kadapa, India; ^3^Department of Microbiology, Yogi Vemana University, Kadapa, India; ^4^Department of Animal Biology, University of Hyderabad, Hyderabad, India; ^5^National Institute of Animal Biotechnology (NIAB), Hyderabad, India

**Keywords:** γδ T cells, pathogenic infections, cancer, autoimmunity, wound healing, immunotherapy

## Abstract

The role of γδ T cells in immunotherapy has gained specific importance in the recent years because of their prominent function involving directly or indirectly in the rehabilitation of the diseases. γδ T cells represent a minor population of T cells that express a distinct T cell receptor (TCR) composed of γδ chains instead of αβ chains. Unlike αβ T cells, γδ T cells display a restricted TCR repertoire and recognize mostly unknown non-peptide antigens. γδ T cells act as a link between innate and adaptive immunity, because they lack precise major histocompatibility complex (MHC) restriction and seize the ability to recognize ligands that are generated during affliction. Skin epidermal γδ T cells recognize antigen expressed by damaged or stressed keratinocytes and play an indispensable role in tissue homeostasis and repair through secretion of distinct growth factors. γδ T cell based immunotherapy strategies possess great prominence in the treatment because of the property of their MHC-independent cytotoxicity, copious amount of cytokine release, and a immediate response in infections. Understanding the role of γδ T cells in pathogenic infections, wound healing, autoimmune diseases, and cancer might provide knowledge for the successful treatment of these diseases using γδ T cell based immunotherapy. Enhancing the human Vγ9Vδ2 T cells functions by administration of aminobisphosphonates like zoledronate, pamidronate, and bromohydrin pyrophosphate along with cytokines and monoclonal antibodies shows a hopeful approach for treatment of tumors and infections. The current review summarizes the role of γδ T cells in various human diseases and immunotherapeutic approaches using γδ T cells.

## Introduction

T cells are the principal lymphocytes that play a vital role in cell-mediated immune responses. Majority of the T cells are αβ T cells with α and β chains and a minor population of γδ T cells do exist that play a pivotal role in the host defense system (1). γδ T cells are located in peripheral blood (PB), intestine, skin, spleen, and lymph nodes where they were found to act as interface for the cross talk between innate and cell-mediated immune cells (2). The functional development of γδ T cells is initiated much earlier than the development of αβ T cells in embryogenesis, where γδ T cell Receptor (TCR) gene rearrangements were detected in the fetal thymus by “day 14” of embryogenesis (E14). The earliest murine γδ T cell “wave” expresses Vγ3Vδ1 and gives rise to skin dendritic epidermal γδ T cells (DETC). Later, vaginal and gut intra-epithelial γδ T lymphocytes (IEL) and immune organ γδ T cells were developed (3). Vγ3Vδ1 DETCs are exclusively generated in the fetal thymus, migrate to epidermal epithelia, expand locally and are maintained throughout the life of an individual (4). γδ T cells express TCR molecule on their cell surface, but there are only a few variable genes available to construct Vγ/Vδ TCR proteins. In addition, the usage of the Vγ/Vδ genes is not random but appears to be dictated by the localization of γδ T cells (5). Hence γδ T cells are well engaged in newborns to contribute to immune-protection and immune-regulation (6, 7).

γδ TCR recognize non-peptide antigens like glycerolipids and other small molecules, polypeptides that are soluble or membrane anchored, and cross linked to major histocompatibility complex (MHC) molecules or MHC-like molecules in an antigen-independent manner (8). Human Vγ9Vδ2 (also known as Vγ2Vδ2) T cells can be activated by metabolites from isoprenoid synthetic pathway. These include (E)-4-hydroxy-3-methyl-but-2-enyl pyrophosphate (HMBPP), exogenous prenyl pyrophosphate from bacteria and parasitic protozoa and isopentenyl pyrophosphate (IPP), and endogenous prenyl pyrophosphate deriving from mevalonate pathway that operates in human. Aminobisphosphonates may activate Vγ9Vδ2 T cells by inhibiting the key enzyme farnesyl pyrophosphate synthase of mevalonate pathway in certain tumors leads to upregulating the endogenous pool of IPP (9). Vavassori et al. identified butyrophilin BTN3A1 molecule, which is involved in the presentation of phosphorylated antigens to Vγ9Vδ2 T cells (10). Recently, Sandstrom et al. demonstrated that intracellular B30.2 domain of butyrophilin 3A1 (BTN3 A1) protein binds phosphoantigens (pAg) to mediate activation of human Vγ9Vδ2 T cells. This intracellular B30.2 domain of BTN3A1 directly binds pAg through a positively charged surface pocket and charge reversal of pocket residues abrogates binding and activation of Vγ9Vδ2 T cells (11). Furthermore, Uldrich et al. reported that CD1d presents lipid-based antigens to human Vδ1^+^ γδ T cells (12). γδ T cells use both the TCR and also additional activating receptors like natural killer (NK) cell activating receptor (NKG2D), toll like receptor (TLR), and NOTCH signaling to respond to stress induced ligands and infection (13). Activated γδ T cells promote the anti-infection capabilities of resident macrophages, NK cells, and also enhance the maturation of dendritic cells (DCs). Besides that, they secrete cytokines and chemokines to recruit pro-inflammatory neutrophils to accelerate the elimination of pathogens and the repair of damaged tissues. Neutrophils in turn suppress the γδ T cells activation to reduce the inflammation when the infection has been resolved (14). In addition, γδ T cells in the skin produce keratinocyte growth factor (KGF), important cytokine for wound repair, and epithelial cell regeneration. It has been demonstrated that the human γδ T cells activation and expansion can be controlled by forkhead box P3 (FOXP3) expressing regulatory T cells (Tregs), programed death-1 (PD-1), and cytotoxic T lymphocyte antigen (CTLA)-4 both *in vivo* and *in vitro* (15).

γδ T cells bridge innate and adaptive immunity and play a protective role in immune-surveillance. Effector γδ T cells produce interferon (IFN)-γ, tumor necrosis factor (TNF)-α, which enhance cell-mediated immune response and interleukin (IL)-17 that plays a vital role in early neutrophil mediated response. In addition, cytotoxic components such as perforin, granzymes secreted by these cells ultimately cause direct or indirect effect of cytotoxicity against infected cells (16). They provide a wide range of defense mechanisms against microorganisms such as viruses, bacteria, protozoa, and diseases like cancer and also in healing of wounds and burns. In addition, γδ T cells also play a role in autoimmune diseases such as rheumatoid arthritis (RA) and systemic lupus erythematosus (SLE) through their antigen-presenting capacity, release of pro-inflammatory cytokines, immunomodulatory properties, interaction with Tregs, and promotion of antibody production (17). Pantelyushin et al. reported that apart from retinoid-related orphan receptor gamma-t (RORγt^+^) innate lymphocytes, γδ T cells also produce cytokines like IL-17A, IL-17F, and IL-22 that are essential and enough for psoriatic plaque formation in a disease model that closely resembles human psoriatic plaque formation (18). Current review exclusively focuses on the role γδ T cells in specific pathogenic infections, anti-tumor activity, healing of wounds and burns, autoimmune diseases, and few insights on their immunotherapy.

## Pathogenic Infections

### Tuberculosis

Tuberculosis caused by *Mycobacterium tuberculosis* (Mtb) is considered to be one of the serious infectious disease worldwide causing 1.7 million deaths every year. Around 30% of the world’s population is affected by *M. tuberculosis* and approximately 100 million people died due to tuberculosis (TB) over the last century (19). Hence, there is an urgent need to find out the host factors that delineate the individuals susceptible to TB. pAg such IPP and HMBPP are the key ligands that activate Vγ9Vδ2 T cells. HMBPP is nearly 1000-fold more effective than IPP for the *in vitro* activation of Vγ9Vδ2 T cells (20). Mtb produces HMBPP, which is recognized by Vγ9Vδ2 TCR and drives the activation of Vγ9Vδ2 T cells (21). Effector Vγ9Vδ2 T cells are shown to participate in the anti-TB immune response by production of various cytokines (Th1, Th2, and Th17) and also activation of other immune cells such as CD4^+^ and CD8^+^ T cells, B cells, DCs, and macrophages (22). The *in vivo* studies have demonstrated that the major expansion of Vγ9Vδ2 T cells in macaques is induced only by HMBPP plus IL-2 co-treatment, but not IL-2 or HMBPP alone (23) although IL-2 treatment of macaques expands CD4^+^CD25^+^Foxp3^+^Treg cells (24). In a primate model for TB, γδ T cells produce IL-22 initially, which can be down regulated by HMBPP. There are various subsets of γδ T cells, which are self regulative, and HMBPP treatment during early stages of infection might be helpful in evading Mtb (25). Peng et al. showed that upon stimulation with Mtb heat treated antigen (Mtb-HAg), levels of IFN-γ producing Vγ9Vδ2 T cells increased in number and were the main source of IL-17 (26). This led to the increased recruitment of phagocytic cells to the infected site and formation of granulomas in pulmonary TB. This reaction was antigen specific, because immunizing the same host once again with Mtb-HAg has led to faster reactivation of Vγ9Vδ2T cells. Thus, stimulation of Vγ9Vδ2 T cells with pyrophosphates like IPP and HMBPP might represent a novel vaccine strategy to identify the key effector pathways of *Mycobacteria* stimulated Vγ9Vδ2 T cells that potentially act to inhibit the intracellular growth of *Mycobacteria*. In addition to that, human Vγ9Vδ2 T cells can recognizes many pathogen antigens and show rapid immune responses during infections including *E. coli* infections, salmonellosis, brucellosis, leprosy, tularemia, legionellosis, and listeriosis (27).

Bovine TB is caused by *Mycobacterium bovis* is a major zoonotic problem in United Kingdom and developing countries. γδ T cells show a major immunological response against *M. bovis* infection. Workshop cluster 1 (WC1) molecule expressed on Vγ9Vδ2 T cells is involved in the antigen recognition including heat-shock proteins, phospholipids derived from *Mycobacteria*, and other non-peptide antigens. WC1^+^ γδ T cells are important in the development of granulomas during *M. bovis* infection by upregulating IFN-γ, IL-12, IL-18, MHC II, CD80/86, CD40, and adhesion molecules (22).

### Malaria

Malaria is a mosquito borne infectious disease caused by parasitic protozoan *Plasmodium falciparum* in humans and other animals. The WHO report 2013, has an estimate of 207 million cases of malaria with approximately 0.627 million deaths and 3.4 billion people prone to the risk of malaria (28). The information about the *in vivo* activation and anti-plasmodial action of the Vγ9Vδ2 T cells is indistinct and it is important to understand the mechanisms of early control of the parasite multiplication and parasite density. It has been shown that elevated levels of γδ T cells in PB and spleen occur during acute plasmodium infection (29). γδ T cell-deficient [TCRδ-knockout (KO)] mice were unable to clear the infected red blood cells, showed high parasitemia and eventually died (30). Therefore, γδ T cells have the potential to react with malaria antigens rather than the αβ T cells or by NK cells (31). Vγ9Vδ2 TCR recognizes schizont associated antigen (SAA) and HMBPP, are the antigens of *P. falciparum* result in the activation of Vγ9Vδ2 T cells (31, 32). Activated γδ T cells produce huge amount of IFN-γ in presence of activated monocyte cytokines IL-10, IL-2, and IL-1β. TLR-sensitized DCs express enhanced co-stimulatory factors on their surface and induce high levels of IFN-γ production by human Vγ9Vδ2 T cells (33). The CD40 ligand on γδ T cells is ligated with CD40 on DCs and this signaling synergistically enhances the uptake of plasmodium antigens via DCs by increasing the production of IL-12 (30). Previous reports suggest that the number of polyclonally activated Vδ2^+^ T cells increase in PB during the acute phase of *P. falciparum* in malaria. In addition, *in vitro* activated Vγ9Vδ2 T cells express granzyme-A and B, perforin, Fas/Fas ligand (FasL) and granulysin to kill the asexual stages of *P. falciparum* in the blood as well as inhibit the growth of intra erythrocytic stages (34). The *P.falciparum* was also known to be inhibited by human Vγ9Vδ2 T cells *in vitro*. The targets recognized by Vγ9Vδ2 T cells are extracellular merozoites of the host erythrocytes and this exclusively requires the contact between γδ T cells and the merozoites (35). Both the blood stages of intra erythrocytic parasite and extracellular merozoites themselves activates the Vγ9Vδ2 T cells resulting in their degranulation by granulysin, but not by perforin (36). Teirlinck et al. reported that Vγ9Vδ2 T cells have the ability to develop effector memory cells after infection with *P. falciparum* (37) and this feature might be helpful in the development of novel cell based malaria vaccine.

### Acquired immunodeficiency syndrome

Acquired immunodeficiency syndrome is caused by human immunodeficiency virus (HIV) and is one of the greatest health crises ever faced by the global community. It has been demonstrated that circulating Vγ9Vδ2 T cells exhibit anti-HIV role by secreting chemokines for HIV entry co-receptors, producing soluble antiviral factors, and killing the infected cells by the mechanisms similar to cytotoxic T lymphocytes (CTL) and NK cells. However, Vγ9Vδ2 T cells are found to be depleted in the advanced stages of the HIV infection and the insufficient number of Vγ9Vδ2 T cells leads to increased potential for chronic inflammation. The envelope glycoprotein 120 (gp120) of CCR5 tropic strains of HIV could bind with the surface receptors CCR5 and α4β7 expressing on γδ T cells. This binding activates the P38 MAP kinase, which in turn promotes the Fas dependent caspase activation and induces the cell death (38). Further, when macaques were immunized with proteins like simian immunodeficiency virus (SIV) gp120 and gag p27 induced the production of chemokines CCL-3, CCL-4, CCL-5 (RANTES), which in turn bind with the CCR5 thus inhibiting the entry of HIV into the host (39). The levels of these chemokines were increased by the engagement of the ectopically expressed receptors called Natural Cytotoxic Receptors (NCRs) like NKP30 on the surface of the Vδ1 T cells that induced the production of high levels of significant chemokines and controlled the levels of HIV (40). However, activated Vγ9Vδ2 T cells may directly suppress HIV replication by releasing CC-chemokines, competing with HIV for CCR5 entry coreceptor, and other soluble antiviral factors (41). Hence, the treatment approaches might include targeting proviral infection using the activation of Vγ9Vδ2 T cells with aminobisphosphonates and IL-2 to improve the anti-viral activity but not on the prolonged virus as the viral load is severe in this condition. γδ T cells coordinate activated innate immunity with adaptive antibody and T cell responses in preventive vaccination against HIV-1 infection.

Apart from HIV, γδ T cells were known to be involved in following viral infections. γδ T cells are highly specific to the micro-environment in which they thrive and said to possess organ specific functions. γδ T cells in liver produce IL-17 is very essential for meliorating adenovirus mediated hepatitis, neutralizing IL-17 with antibodies aggravated these conditions (42). γδ T cells can compensate neutrophil IL-17 production. For instance, when mice were infected with *Cryptococcus neoformans* strain-H99γ, which leads to neutropenia, IL-17 producing γδ T cells mediated the regulation of innate and adaptive cells to mount a successful immune response (43). γδ T cells were known to possess memory just like other adaptive immune cells. This character of γδ T cells was brought into limelight by the association of γδ T cells response on cytomegalovirus (44). γδ T cells were reduced in number during sepsis and acute reduction of these cells resulted in mortality of patients suffering with sepsis (45). The protective role of γδ T cells have also been confirmed in few other infectious diseases caused by viruses including influenza virus, West Nile virus, herpes simplex virus, Epstein–Barr virus, and human hepatitis virus C.

## Cancer

**Figure 1:**
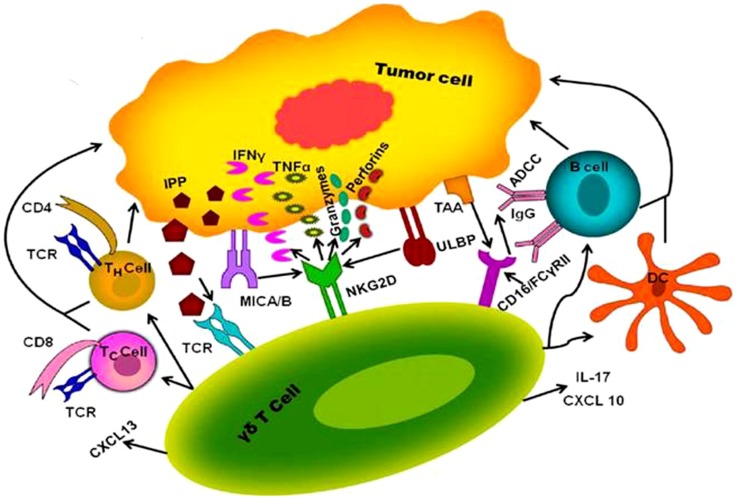
**Proposed mechanism of interaction between human γδ T cell and tumor cell**. γδ T cells activated through interaction of γδ T cell receptor with IPP produced via mevalonate pathway at higher concentration in tumor cell. Besides that NKG2D provides activation signals upon binding to MICA/B and ULBP of tumor antigens results in the release of cytokines and chemokines such as IFN-γ, TNF-α, IL-2, IL-17, perforin, grazymes, CXCL-10, and CXCL-13, which can directly lyse the tumor cell and can recruit other immune cells like T cells (T_H_ cells and T_C_ cells), B cells, and dendritic cells (DC) to aid killing of tumors. γδ T cells expressing CD16 receptor (FCγRIII) interacts with TAA mediate ADCC through activation of B cell.

γδ T cells have a unique role in the immune-surveillance against malignancies and also an advantage over αβ T cells because they can directly recognize molecules that are expressed on cancer cells without need of antigen processing and presentation (46). An important therapeutic feature of γδ T cells is that these favorably kill cancer cells and show low reactivity toward non-transformed cells (47). Variable region of the Vγ9Vδ2 TCR plays a major role in recognition of the antigen (48). Vγ9Vδ2 TCR recognize increased pool of endogenous IPP, which may only be found in tumor cells but not in healthy tissues. Activated Vγ9Vδ2 TCRs promote γδ T cell cytotoxicity through increased secretion of perforin/granzymes, IFN-γ, and TNF-α, IL-17, up-regulates expression of FasL and TNF-related apoptosis-inducing ligand (TRAIL) (49). Among the list of mediators promoting anti-cancer cytotoxicity mentioned above, IL-17 in tumor micro-environment remains controversial showing anti-tumor role and pro-tumor role. In several murine transplantable tumor models, anti-cancer drugs (such as oxaliplatin or anthracyclines) that induced immunogenic cell death, triggered the local invasion of IL-17 producing γδ T cells, which occurred before and was required for the subsequent invasion of tumor-reactive CTL (50, 51). However, it was shown that IL-17 producing γδ cells acts as tumor promoting cells by inducing angiogenesis (52). In addition, Rei et al. demonstrated that IL-17 producing Vγ6^+^ γδ cells promotes tumor growth in the ID8 ovarian cancer model and thus opposes the widely accepted anti-tumor function of γδ cells (53). In addition to TCR dependent pathways, NKG2D and DNAX accessory molecule-1 (DNAM-1) expressed on Vγ9Vδ2 T cells plays a critical role in anti-tumor response as shown in the Figure 1. NKG2D provides activation signals upon binding to non-classical MHC molecules of the MHC class-I chain-related molecules (MICA/B) and UL-16 binding protein (ULBP) families expressed on tumor cells (54). Nectin-like-5 expressed in carcinoma cells recognized by DNAX and provides activation signals (50). γδ T cells also express CD16 (FCγRIII) receptor, upon recognition of tumor associated antigens (TAA), CD16/FCγRIII receptor binds to Fc portion of immunoglobulin G (IgG) and leads to antibody-dependent cellular cytotoxicity (ADCC) (55). In addition to the cytokines, cytotoxic soluble factors, activated Vγ9Vδ2 T cells can produce large amount of chemokines like CXCL-13 and CXCL-10 result in the recruitment of B cells, macrophages, T cells, and NK cells toward the tumor micro-environment (49). It leads to inhibition of tumor growth and reduced survival of autologous tumor cells. γδ T cells have a unique capacity to present antigens to both CD8 and CD4 T cells and potentially elicit strong adaptive anti-tumor response.

Presently two strategies were applied for γδ T cell based cancer immunotherapy. One is the adoptive cell transfer of *in vitro* expanded Vγ9Vδ2 T cells and second one is the administration of pAg or aminobisphosphonates along with low-dose recombinant IL-2 to stimulate Vγ9Vδ2 T cells *in vivo*. It has been shown that the two synthetic molecule drugs bromohydrin pyrophosphate (BrHPP) and zoledronate selectively activate human Vγ9Vδ2 T cells in clinical trials. Bioactivity (EC_50_) of BrHPP (56, 57), and zoledronate (58) were 24 nM and 1 μM, respectively to activate TCR Vγ9Vδ2 T cell clones and PB mononuclear cells *in vitro*. Dieli et al. reported that increased survival rates in prostate cancer patients when administered zoledronate (4 mg) in combination with IL-2 (0.6 × 10^6^ IU) was observed (59). In another clinical trial (60), administration of low-dose IL-2 (0.25 to 3 × 10^6^ IU) in combination with pamidronate (90 mg) in multiple myeloma patients showed that five out of nine patients had significant *in vivo* activation and proliferation of Vγ9Vδ2 T cells, indicating that Vγ9Vδ2 T cells might contribute to this anti lymphoma effect. Vγ9Vδ2 T cells from tumor infiltrate lymphocytes (TIL) of colorectal cancer are able to eliminate cancer cells *in vivo* and have been associated with reduced metastasis and longer mean survival (61). BrHPP activated TCR Vγ9^+^ γδ T cells obtained from healthy subjects co-incubated *in vitro* with B-lymphoma cell targets coated with rituximab, enhance ADCC of target cells (62). In primates, administration of pAg synthetic analogs and low doses of IL-2 expand Vγ9Vδ2 T cells in lung tissue, which in turn confer activity against human non-small cell lung cancer (NSCLC) cell lines by increasing the secretion of IFN-γ, TNF-α, and TRAIL (63). Phospho Ag-expanded Vδ2 T cells infusion has also been tested in lymphoid neoplasms and renal cancer. In metastatic renal carcinoma, six cycles of adaptive immunotherapy with infusions of phospho Ag-expanded γδ T cells plus zoledronic acid (4 mg) and low doses of IL-2 (1.4 × 10^6^ IU) caused complete remission without progression for 2 years (64). *In vitro* studies reported γδ T cells killing efficacy was increased against three cervical cancer cells pretreated with pamidronate and proved by LDH cytotoxicity test (65). Elevated levels of Vγ9Vδ2 T cells in the liver were found in hepatocellular carcinoma (HCC) patients as well as tumor bearing mice compared to healthy controls, indicating the importance of Vγ9Vδ2 T cell in the anti-tumor immunity (66). Oberg et al. reported increased γδ T cell cytotoxicity against pancreatic ductal adenocarcinomas (PDAC) *in vitro* and *in vivo* in immunocompromised mice, when administered healthy donors γδ T cell with Her2/Vγ9 bispecific antibodies (67). Hence, novel regimens combining γδ T cells with drugs or monoclonal antibodies would be helpful for treatment of solid tumors and hematological malignancies.

## Burns and Wounds

γδ T cells participate in several aspects of healing from burn injuries and wounds. Healing is a dynamic and complex process requiring constant communication between cells in the form of cytokine release, cell-to-cell contacts, and cell-to-matrix interactions. The epidermis is a barrier tissue that is exposed to the environment and susceptible to injury. γδ T cells are found in both epidermis and dermis of human skin express Vδ1 chain. These skin-resident T cells involved in reepithelization of acute and chronic wounds. Human epidermal αβ and Vδ1 bearing T cells are able to produce insulin like growth factor upon activation and promote wound healing in a skin organ culture model (68). Murine epidermal γδ T cells are named as DETCs because of their unique dendritic morphology. DETC express an invariant Vγ3Vδ1^+^ TCR. Oppeltz et al. reported that epidermal γδ T cells play a major role in the expression of inducible nitric oxide synthase (iNOS) at the burn wound site and is important in wound closure and collagen deposition (69). Recently, it has been suggested that DETC participate in tissue repair, likely through the production of Th2 and Th17 cytokines (70), chemokines, and growth factors. Hence the immediate goal of DETC is to repair wound and maintain tissue integrity and homeostasis.

**Figure 2:**
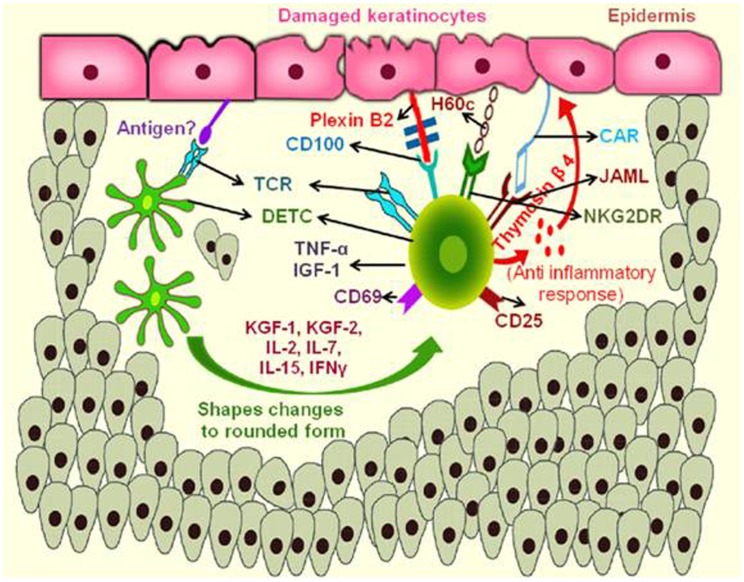
**Mechanism of action of murine skin resident γδ T cells in wound repair**. During keratinocyte damage, the epidermal γδ T cells (DETCs) are activated upon recognition of an unknown antigen, rounding of the DETCs occur and produce thymosin β4 and cytokines like TNF-α, IFN-γ, and IL-2 required for anti-inflammatory response and wound healing. Engagement of NKG2DR with H60c and JAML–CAR interactions also enhances the production of these cytokines and provide epidermal repair. (JAML, junction adhesion molecule like protein; CAR, coxsackie and adenovirus receptor; TNF, tumor necrosis factor; IFN, interferon; IL, interleukin; NKG2DR, natural killer cell activating receptor).

Wound occurs as a result of trauma, infection or by the pathological infections. Cellular damage and stressed keratinocytes produce an unknown antigen, which binds with the TCR of DETC, which are proximal to the wound (71). Further, DETC changes its morphology and become rounded with in 4 h post wounding (72). The rounded morphology of DETC correlates with functional activity. The distal DETC to the wound retain dendritic and maintain tissue homeostasis. Activated DETC produce TNF-α, insulin growth factor (IGF)-1, KGF-1, KGF-2, and up regulate the activation markers CD25 and CD69 in response to the epithelial damage (73). Rapamycin, an immunosuppressor that is known to regulate the DETC rounding by targeting the serine threonine kinase, cause delay in cytokine production and wound healing (74). DETCs are shown to produce lymphoid associated thymosin-β4 variant in contact dermatitis that exhibits the anti-inflammatory role (75). In normal condition, DETCs are slightly activated at the epidermal region by expressing the activation markers CD25, CD69, and also secrete some cytokines in low amount that allows them for quick activation in response to local trauma (76). DETC express the co-stimulatory activating receptors like NKG2D, and H60c, ligand expressed on wounded epidermis and shown to provide co-stimulatory signals with NKG2D participating in TCR mediated signaling (77). A prominent signaling by co-stimulatory mode of DETC activation is by junctional adhesion molecule-like protein (JAML) that is expressed by the DETC. The recognition of JAML to its ligand coxsackie and adenovirus receptor (CAR) expressed by the keratinocytes results in the recruitment of phosphoinositide 3-kinase (PI3K) (78) and also with the HLA4E10 (79) stimulatory antibody that helps in promoting wound healing as shown in Figure 2. Recently, it was found that plexin B2 expressed on keratinocytes and CD100 an intra cellular signaling domain of DETCs shows much interaction and makes the cellular rounding via signals through ERK kinase and cofilin (80). Rani et al. reported that, γδ T cells regulate myeloid cell activity, which in turn enhances macrophage influx into the wound site to repair wound (81).

γδ T cells also participate in the repair of epithelia in other organs, such as intestine, lung, and cornea. γδ T cells play a key role in the maintaining of intestinal mucosa by producing KGF. Mice lacking γδ T cells treated with a dextran sodium sulfate (DSS), exhibit severe mucosal injury and decreased epithelial cell proliferation (82). Mice deficient for TCR δ chain (TCRδ^−/−^) exhibit significantly reduced inflammation and epithelial repair in the lung after bleomycin treatment (83) and also in the corneal epithelial abrasion (84). In the lung injury, γδ T cells are required for the neutrophils influx and also produce IL-17, which are responsible for the inflammatory response and epithelial repair (85). Corneal healing involves both epithelium and sensory nerves that have a trophic effect for the epithelium. Intracellular cell adhesion molecule (ICAM)-1 and CCL20 chemokine are necessary for attracting the γδ T cells into the healing epithelium (86). γδ T cells are responsible for the secretion of cytokines such as IL-17, IL-22, and the influx of neutrophils and platelets, which contribute to the epithelium healing. Accumulation of neutrophils and platelets leads to the rise in vascular endothelial growth factor (VEGF), which is required for nerve regeneration (87).

## Autoimmune Diseases

Autoimmune diseases are characterized by abnormal immune responses to self antigens. These diseases are induced by many environmental factors on a genetically susceptible background, which lead to production of huge number of inflammatory cytokines and auto-antibodies to make path for the outbreak and progression of the disease. The roles of γδ T cells in autoimmune diseases are not yet very clear. Bendersky et al. showed that elevated number of Vγ9Vδ2 T cells (~35%) in synovial fluid and PB, produced TNF-α and IFN-γ during the pathogenesis of juvenile idiopathic arthritis (JIA). Further, activation of γδ T cells leads to apoptosis of synovial fibroblasts, the effect of which is manifested in JIA (88). γδ T cells in intestine were significantly reduced during intestinal inflammation, which leads to the uncontrolled activity of CD4^+^ T helper cells to cause autoimmune inflammatory bowel disease called Crohn’s disease (89). The role of γδ T cells in allergic encephalomyelitis and multiple sclerosis is ambiguous. γδ T cells were certainly abundant in cerebrospinal fluid of the respective mouse models but their precise role is not yet established (90). In non-obese diabetic mouse model, γδ T cells infiltrate into islet cells and secrete IL-17 to cause apoptosis. Antibody mediated blockade of IL-17 stopped the process and rescued mouse from diabetes (91). γδ T cells cross react with aminoacyl-tRNA synthetases of the muscle cells that lead to the pathogenesis of myositis, an inflammation of muscle tissues caused by autoimmune T cells (92).

Behcet’s disease is an immune mediated disorder that is often associated with the symptoms like ulceration of mucous membrane, skin, and ocular problems. In general, normal individuals consist of CD45RA^−^ CD45RO^+^ Vγ9^+^Vδ2^+^ T cells but as the disease progress abnormal γδ T cells with CD45RA^+^ CD45RO^−^ Vγ9^+^Vδ2^+^ phenotype are increased in number and activated, then produced IL-2R in patients suffering with Behcet’s disease (88). Infliximab, is a chimeric monoclonal antibody against TNF-α, suppress the Vγ9Vδ2 T cell expansion and activation by blocking the TNF receptor on Vγ9Vδ2 T cells. Moreover, it also reduces the cell-mediated immune responses by reducing the expression TNF RII, perforin, granzyme-A, and IFN-γ production from Vγ9Vδ2 T cell (93).

Psoriasis is speculated as the T cell operated chronic inflammatory skin disease (94), but the mechanisms of pathogenesis are still poorly understood. Chemokine receptor CCR6 (receptor for a CCL20) is very abundant in psoriatic skin T cells. Chemokine receptors are transmembrane proteins that are activated by chemokines, which play key roles in cell trafficking, cell motility, and survival. CCR6 regulates epidermal trafficking of γδ T cell subsets in the skin (95). The chemokine receptor CCR6 is expressed on the Th17 cells and γδ T cells, which produce cytokines like IL-17 and IL-22, TNF-α, and IL-20 (94). IL-22 plays a key role in the activation of immunity and has been implicated in the pathogenesis of psoriasis (96). γδ T cells from CCR6 KO mice not only failed to accumulate in the epidermis after IL-23 treatment and even though entered into the epidermis produced low amounts of IL-22 compared with wild type γδ T cells. Hence, this data suggest that not only recruitment but also function of γδ T cells may be impaired in the absence of CCR6 (97). Laggner et al. showed that, redistribution of Vγ9Vδ2 T cells from PB to skin in psoriasis patients as compared to the healthy control. These skin homing Vγ9Vδ2 T cells produce the psoriasis relevant cytokines IFN-γ, TNF-α, and IL-17A similar to blood derived Vγ9Vδ2 T cells (98, 99). Prominent roles for skin homing Vγ9Vδ2 T cells are influencing the resident immune and epithelial cells by the rapid release of pro-inflammatory cytokines, the recruitment of immune cells from the circulation, and in tissue remodeling by the release of growth factors (99), which make Vγ9Vδ2 T cells as the biomarker of psoriasis.

γδ T cells show enhanced antigen-presenting cell (APC) functions, which may play a role in the pathogenesis of RA by over activating T and B cells. Abnormal activation of Th1 and Th17 cells result in the production of pro-inflammatory cytokines, which play crucial role in the pathogenesis of RA (100). Peripheral Vγ9Vδ2 T cells after stimulation with IPP *in vitro* are shown to upregulate the expression of APC specific molecules HLA-DR and CD80/86 and presented soluble antigens and synthetic peptides to CD4^+^ T cells and B cells thus contributing to activation of CD4^+^ T cells and being associated with RA onset and disease progression (100). A subset of CD27^+^ CD25^high^ Vδ1 T cells expressing FoxP3 were gradually decreased in the PB of systemic lupus erythematosus patients progressing in the pathogenesis of SLE and these regulatory γδ T cells could be generated *in vitro* when stimulated with anti γδ-TCR in presence of IL-2 and transforming growth factor-beta (TGF-β) (101). The activating marker CD69 and HLA-DR were up regulated while the expression levels of the inhibiting receptor CD94/NKG2A remained low after the antigen stimulation on these γδ T cells, upon activation might lead to the over activation of γδ T cells in patients with SLE (102).

Graft rejection is a serious problem during transplanting of solid organs. Conventionally, the B cells and αβ T cells of adaptive immune system were considered to be the key in this phenomenon as they have immune memory and can bind numerous antigens. However, there is an evidence that γδ T cells play a vital role in graft rejection. Gorczynski et al. studied the role of γδ T cells in skin graft rejection using mouse model (103). γδ T cells were not restricted by immunosuppressive drugs during organ transplantation. Some subsets of γδ T cells expand oligo clonally and the reasons for this expansion are unknown but may be linked to persistent viral infections. It has been suggested that quantification of γδ T cells in PB may not be essential to decide graft tolerance (104). The known role of γδ T cells in autoimmune diseases is limited, so an extensive research has to be focused particularly on the effects of these cells in the pathogenesis and development of disease and ultimately for the development of γδ T cell based therapies.

γδ T cells functions in various diseases, described in the review are summarized in the Table 1.

**Table 1:** **Antigen recognition and functions of γδ T cells in various diseases**. γδ T cells recognizes various antigenic factors and produce respective chemokines and cytokines to protect immune system against from different diseases like pathogenic infections, cancer, wound repair, and autoimmune.

S. No.	Disease	Stimulator/activator for γδ T cells	Function of γδ T cells in immune-protection
1.	Pathogenic infections
	a. Tuberculosis (*M. tuberculosis*)	HMBPP produced by the 2-C-methyl-D-erythritol 4-phosphate (MEP) pathway in microorganisms	Produce IL-22, IL-17, and IFN-γ. Regulating both innate and adaptive immunity
	b. Malaria (*P. falciparum*)	Schizont associated antigen (SAA) and HMBPP	IL-10, IL2, IL-1β, and IFN-γ degranulation of infected RBC and merozoites
	c. AIDS (*HIV*)	Recognize the envelope protein GP 120 by CCR5 receptor on Vγ9Vδ2 T cells	Activates P38 MAP kinase, which promotes the FAS dependent caspase activation and induces the cell death
2.	Cancer	Vγ9Vδ2 TCR recognizes endogenous IPP, produced by mevalonate pathway in tumor cells	Increased secretion of perforin/granzymes, TNF-α, IFN-γ, and suppress the tumor
		NKG2D expressed on γδ T cells recognizes MICA/B and ULBP families expressed on tumor cells	Produce IFN-γ, IL-17, and chemokines, which recruit the macrophages, NK cells, B cells, and T cells
3.	Wound repair	Non-specific antigen recognition by DETC	IL-2, TNF-α, IFN-γ, KGF-1, and KGF-2 are produced against the damaged keratinocytes result in the healing of wound
4.	Autoimmune diseases	Recognizes self antigens by γδTCR	Enhances the production of IL-17, IL-23, and IFN-γ. The exact mechanism of γδT cells in these diseases is not yet clear

*HMBPP, (E)-4-hydroxy-3-methyl-but-2-enyl pyrophosphate; γδ TCR, γδ T cell receptor; IFN, interferon; IL, interleukin; GP 120, glycoprotein; CCR, chemokine receptor; MAP, mitogen activated protein; NKG2D, natural killer cell activating receptor; MICA/B, MHC class-I chain-related molecule; ULBP, UL-16 binding protein; TNF, tumor necrosis factor; DETC, dendritic epidermal γδ T cell; KGF, keratinocyte growth factor*.

## Immunotherapy

Immunotherapy has become an increasingly attractive option for the treatment of cancer (105). γδ T cells may be an excellent target for modulation of immune responses in human diseases. Enhancing γδ T cell functions may open the possibility to formulate new immunotherapeutic regimens, which could impact the improvement of immune control of various diseases. Aminobisphosphonates like zoledronate, pamidronate, and BrHPP were well-studied among the numerous activators of Vγ9Vδ2 T cells. IPP is an intermediate metabolite of mevalonate pathway. Bisphosphonates may activate Vγ9Vδ2 T cells, by inhibiting the key enzyme farnesyl pyrophosphate synthase of mevalonate pathway in certain tumors leads to upregulating the endogenous pool of IPP. Mevalonate is the product of HMG-CoA reductase, a rate limiting enzyme subject to tight regulation. However, high exogenous mevalonate concentration would bypass normal regulation and indirectly stimulates Vγ9Vδ2 T cells by increasing endogenous IPP levels (106). Taken together, bisphosphonates and mevalonate may activate Vγ9Vδ2 T cells and aid in curing bacterial infections (107). Bisphosphonates were proven to treat gliomas (108). A clinical trial of immunotherapy using a combination of zoledronate, a bisphosphonate, and IL-2 was found to alleviate renal cell carcinoma (109). HMBPP is one of the key ligands that activate Vγ9Vδ2 T cells. HMBPP is produced by the 2-C-methyl-d-erythritol 4-phosphate (MEP) biosynthetic pathway in microorganisms. HMBPP is nearly 1000-fold more effective than IPP for the *in vitro* activation of Vγ9Vδ2 T cells. Vγ9Vδ2 T cells express surface marker CCR5, which is recognized by gp120 of HIV. This leads to apoptosis of HIV infected cells through a caspase mediated pathway. It has been suggested that by triggering rapid proliferation of Vγ9Vδ2 T cells may help in curing HIV infection (110). γδ T cell based immunotherapy might have a beneficial effect in patients with chronic hepatitis C virus infection (111).

αβ T cell populations potently attack specific targets but are limited by their specificity, where as γδ T cells in combination with tumor-targeting antibodies might provide anti-tumor cytotoxic effects and also long-lasting protection upon antigen presentation. γδ T cells have dual role of stimulating both γδ T cell-directed anti-tumor activity and antigen-specific CD4 and CD8 αβ T cell responses. γδ T cells are attractive agents for cancer immunotherapy because they are not MHC restricted like conventional T cells. So, a single vaccine can be used in all individuals regardless of MHC haplotype (112). Memory like expansion of Vγ9Vδ2 T cells has been reported in primates after subclinical systemic infection and reinfection with attenuated *Listeria monocytogenes* strains (20). γδ T cells acquire memory in order to play protective antiviral and anti-tumor roles. The direct or indirect stimulation of γδ T cells by TLR agonists could be a strategy to optimize Th1 mediated immune responses as adjuvant in clinical trial of cancer immunotherapy (113). Recent studies suggest that, combination of γδ T cells with therapeutic monoclonal antibodies can efficiently mediate ADCC against tumors (114). Chronic wounds are an increasing clinical problem, understanding advanced mechanisms of DETC during wounds and burns in humans suggesting possible therapeutic targets for tissue repair and skin homeostasis. Disabling γδ T cells at the specific site by using monoclonal antibodies is a viable therapeutic option for the treatment of autoimmune diseases. Peters et al. reported immunosuppression of γδ T cells by using anti-CD28 mAb and antagonized by TLR-2 ligands (113). Suppressive γδ T cells could have major therapeutic potential for the control of autoimmunity or allergic reactions. Enhancing the activity of Tregs, which inturn secrete PD-1 and CTLA-4 to suppress the activity of γδ T cells, could improve treatment for the autoimmune diseases (115). Modulation of these immunosuppressive check points may be interesting clinical trial in the γδ T cells based immunotherapy.

## Concluding Remarks and Future Directions

The role of γδ T cells in immunotherapy has gained specific importance due to their prominent function involving directly or indirectly in the rehabilitation of the diseases. γδ T cells act as connecting bridge between both the innate and adaptive immune responses without the antigen presentation and producing some important key cytokines like IFN-γ and TNF-α. When stimulated with the combination of aminobisphosphonates and IL-2 activate the Vγ9Vδ2 T cells further and they can also act as the APCs to treat particular infections and tumors. Factors that potentiate the pathogenesis of γδ T cells in autoimmune diseases are yet to be revealed so as to develop drugs, which can target the γδ T cell in particular. New strategies and models that target the molecules involved during disease onset and progression need to be developed, as this makes a pavement for γδ T cell based immunotherapy. New ligands and chemokines responsible for activation and effector functions of γδ T cells are to be deciphered. Many new molecules and proteins can be designed to trigger the γδ T cell through a TCR independent pathway. Vaccines have to be developed to enhance the production levels of chemokines and cytokines by these γδ T cells at the tumor site. For the preparation of large number of cells for adoptive cell transfer, it is necessary to develop better antigens, which stimulate γδ T cell expansion *in vitro*. Further, clinical trials are required for the γδ T cell targeted immunotherapy in case of chronic infections and diseases. Further research might shed more light on the in depth understanding of the underlying mechanisms of the antigen recognition and key factors influencing the γδ T cell production during the disease.

## Conflict of Interest Statement

The authors declare that the research was conducted in the absence of any commercial or financial relationships that could be construed as a potential conflict of interest.
